# Culture Modulates the Neural Correlates Underlying Risky Exploration

**DOI:** 10.3389/fnhum.2019.00171

**Published:** 2019-05-29

**Authors:** Yang Qu, Lynda C. Lin, Eva H. Telzer

**Affiliations:** ^1^School of Education and Social Policy, Northwestern University, Evanston, IL, United States; ^2^Institute for Policy Research, Northwestern University, Evanston, IL, United States; ^3^Department of Psychology, University of Illinois, Urbana-Champaign, IL, United States; ^4^Department of Psychology and Neuroscience, University of North Carolina at Chapel Hill, Chapel Hill, NC, United States

**Keywords:** acculturation, culture, neuroscience, risk taking, fMRI

## Abstract

Most research on cultural neuroscience focuses on one dimension of culture: group membership or individual orientation. However, it is especially important to examine the intersection between the two to better understand the acculturation process. To examine the role of culture in the neural correlates of risky exploration, the current study recruited 22 American and 24 Chinese international students. Participants reported on their independent self-construal, a measure defining the self in terms of emphasizing unique attributes, and underwent an functional magnetic resonance imaging (fMRI) scan while completing a risk-taking task. At the group level, American (vs. Chinese) participants showed greater risky exploration on the task. Moreover, while independent self-construal was not related to American individuals’ behavioral performance and neural correlates of risky exploration, Chinese participants who reported greater independent self-construal recruited greater activation in regions of the cognitive control system [e.g., dorsolateral prefrontal cortex (DLPFC)] and affective system [e.g., anterior insula (AI)], which was related to greater risky exploration. Taken together, our findings suggest that culture as group membership and individual orientation may interact with each other and relate to neural systems underlying risky exploration. This study highlights the importance of studying the role of culture at both group and individual level, which is particularly critical to understand individuals as they acculturate to a new environment.

## Introduction

Most research on cultural neuroscience focuses on one dimension of culture: group membership (e.g., nationality or country of origin) or individual orientation (e.g., self-construal or cultural beliefs) (e.g., Cheon et al., 2011; Wang et al., 2013, 2017). Few studies examine the intersection between the two, partly because the underlying assumption is that individual orientation (e.g., self-construal) explains national or group differences (e.g., Western individuals have high levels of independent self-construal, whereas East Asian individuals have high levels of interdependent self-construal) (e.g., Markus and Kitayama, 1991). However, this assumption may not be valid when studying acculturation. As proposed by Kitayama and colleagues (Kitayama et al., 2006; Kitayama and Park, 2010; Kitayama et al., 2014), voluntary immigrants moving to the frontiers (i.e., new territory in the original country or foreign countries) have greater independent self-construal than those who choose to stay in their original regions. Based on this voluntary settlement hypothesis, individuals who are self-motivated to move to the United States for education and living may show the same level of independent self-construal as their American counterparts (e.g., Cross, 1995; Coon and Kemmelmeier, 2001), although they may still show group-level differences in other domains, such as risk perception or risk behavior (e.g., Brindis et al., 1995; Ojeda et al., 2008; Prado et al., 2009; Almeida et al., 2012; Salas-Wright et al., 2016). Therefore, independent self-construal reported by individuals who immigrate to the United States and American individuals may function in different ways across the two groups. Thus, it is important to examine how independent self-construal (i.e., culture as individual orientation) functions across cultural groups (i.e., culture as group membership), in order to better understand the intersection of culture across two dimensions (group level and individual orientation).

Culture plays a critical role in the motivation to take risks. For example, adventure and risky exploration are highly encouraged by American culture (Doyle, 1999). In contrast, such appreciation of exploration is not evident in East Asian countries, where youth are encouraged to be more inhibited and prudent (King and Bond, 1985; Ho, 1986; Chen et al., 1992, 1998). This may lead American and East Asian individuals to show different behavioral strategies in risk taking. American individuals may exhibit more exploratory behavior in risk-taking contexts, showing a more flexible pattern to test the limits in their exploration, compared to East Asian individuals, who may show a more rigid pattern without much exploration. When East Asian individuals acculturate to a new culture, such as those who move to the United States, individual cultural orientation (e.g., independent self-construal) may play an important role in their risky exploration. For example, those who have a more independent self-construal (i.e., emphasizing internal attributes and preferences) may be willing to explore the environment, potentially leading them to migrate to a foreign country (Heitmueller, 2005; Jaeger et al., 2010) and guiding them to show more exploratory behavior in risk-taking contexts like Americans. In contrast, those who are less acculturated to American culture, as indicated by having less of an independent self-construal, may show less risky exploration.

Recent neuroimaging research on Western samples has provided valuable insights into the motivation of risk taking. Two neural systems—affective and cognitive control systems—have been consistently involved in risk taking in adults and adolescents. The affective system includes regions that code for reward-value and affective salience, including the ventral striatum, ventromedial prefrontal cortex, amygdala, and the anterior insula (AI). For example, greater activation in the ventral striatum, which is involved in reward sensitivity and sensation seeking, is related to more risk-taking behavior, such as drinking, smoking, and drug use (Delgado et al., 2000; Knutson et al., 2000; Galván et al., 2007; Telzer et al., 2013; Qu et al., 2015b). Moreover, the AI has been identified as a hub for the integration of affective and cognitive neural signals (Smith et al., 2014; van Duijvenvoorde et al., 2015) and is recruited when making decisions under uncertainty (Singer et al., 2009; Van Leijenhorst et al., 2010).

The cognitive control system, which includes anterior cingulate cortex (ACC), the dorsolateral prefrontal cortex (DLPFC) and ventrolateral prefrontal cortex (VLPFC; Miller and Cohen, 2001), plays a key role in regulating affective response including risk-related behavior (Fecteau et al., 2007; Cohen and Lieberman, 2010; Schonberg et al., 2012; Wessel et al., 2013). For example, prior research on Western samples has found that risk taking is associated with altered activation in the cognitive control system, including the DLPFC and VLPFC (Schonberg et al., 2012; McCormick and Telzer, 2017). Moreover, disruption of the cognitive control system has been found to involve real-life negative outcomes, such as self-regulatory failure (Heatherton and Wagner, 2011), drug addiction (Goldstein and Volkow, 2011), and unhealthy food choice (Camus et al., 2009), which has important implications for health across the life span.

No extant research has examined the neural processes involved in risk taking among individuals who are acculturating to Western culture. Moreover, prior research on cultural differences in risk perception and risk performance relies on self-reported measures (e.g., Weber and Hsee, 1998; Ojeda et al., 2008), which are unable to capture the dynamic risky exploration and examine whether it varies across cultures. To address this issue, we aimed to examine the role of culture in risky exploration at behavioral and neural levels, which can provide a motivational account to understand risky exploration in different cultural groups. More importantly, to obtain a comprehensive perspective of culture in risky exploration, we conceptualize culture at two levels: group membership and individual orientation. To this end, we recruited both American and Chinese international students. To address the limitation of prior self-reported studies and quantify individuals’ dynamic exploration, participants completed a risk-taking task during a functional magnetic resonance imaging (fMRI) scan. They also reported on their independent self-construal. We first examined group-level differences in behavioral performance and the neural correlates of risky exploration between American and Chinese participants. We next tested whether self-reported independent self-construal differentially relates to risky exploration in the two groups at the behavioral and neural levels.

## Materials and Methods

### Participants

Forty-six first-year undergraduate students participated in the current study, including 22 American (11 female, *M* = 19.19 years) and 24 Chinese (14 female, *M* = 19.59 years) participants. All American participants were born in the United States and self-identified as White. All Chinese participants were international students who were born in China and had been in the United States for less than 1 year prior to their participation. Despite individual variation, first-year Chinese international students typically arrive within 2 weeks before the beginning of the academic year. Therefore, the average time they had been in the United States was between 6–8 months (mean = 7 months, SD = 0.78 month). In addition to specific cultural criteria, participants were also screened for scanner compatibility (right-handed, free of psychiatric disorders, neurological disorders and magnetic resonance imaging (MRI) contraindications). Participants would be excluded from neuroimaging analyses due to excessive head motion during the scan (i.e., relative slice-to-slice movement >2 mm), but no participant showed excessive head movement. Participants provided written consent and all procedures were in accordance with the University’s Institutional Review Board.

### Procedures

Participants completed self-report measures and an fMRI scan. All instructions and stimulus materials were translated and then back-translated from English to Chinese by bilingual speakers (Brislin, 1980). Chinese participants completed the task and all questionnaires in Chinese. A native Mandarin speaking experimenter conducted the study for all Chinese participants.

#### Self-Reported Independent Orientation

Independent self-construal was assessed using the Singelis (1994) self-construal scale (SCS). Participants completed 15 items to rate the extent to which they agree with (1 = *strongly disagree*, 7 = *strongly agree*) a variety of behaviors and attitudes (e.g., “I enjoy being unique and different from others in many respects.” and “I do my own thing, regardless of what others think.”). This measure has been widely used in previous studies to assess the independent self-construal (e.g., Kitayama et al., 2014; Wang et al., 2017). Following previous studies (Kitayama et al., 2014; Wang et al., 2017), the average of all items was taken, with higher scores indicating greater independent self-construal (*α* = 0.72).

#### fMRI Task

To examine neural sensitivity during risk taking, participants underwent an fMRI scan while completing the Balloon Analogue Risk Task (BART). Behavioral performance on the BART is associated with actual risk behaviors such as smoking, addiction, and drug use (Lejuez et al., 2003, 2007; Aklin et al., 2005), suggesting that this task is an ecologically valid measure of real-life risk taking. Furthermore, the BART is widely used in neuroimaging studies to examine neural sensitivity during risk taking (Chiu et al., 2012; Schonberg et al., 2012; Galván et al., 2013; Telzer et al., 2014).

Participants completed the BART during an approximately 9-min self-paced run. Prior to the scan, participants were told that they could earn prizes based on how many points they earned on the BART and participants were instructed to try to earn as many points as possible during the task. During the scan session, participants were shown a series of 24 virtual balloons. By pressing one of two buttons, they can choose either a risky option (i.e., pump the balloon), which results in bigger monetary rewards but a greater probability of getting no rewards (i.e., explosion of the balloon), or a safe option (cash out current rewards). For each successful pump, participants earned one point, and if they cashed out before an explosion, they received the total points earned for that balloon. However, if the balloon exploded before cashing out, participants received no payoff for that balloon. Each balloon exploded at a set level (range = 4–10 pumps; each maximum size appeared three times). Participants were not made aware at any point of the pump levels at which point balloons could explode, which models the unpredictable rewards and punishments of real-world risky behaviors. After each pump, the balloon image disappeared for a jittered interval of 500–4,000 ms before the outcome (e.g., either a larger balloon or an exploded one) was shown on the screen. The payoff for each trial accumulated, as demonstrated by a points meter that remained on the screen, and participants received the total payoff at the end of the task.

We assessed two behavioral indices on the BART. The first is overall risk-taking behavior, which is measured by the number of pumps before cash-outs, with a greater number of pumps before cash-outs indicating greater overall risk-taking behavior. Following prior studies, we did not analyze the number of pumps in explosion trials, because the pumps on the explosion trials were necessarily constrained by the pre-determined maximum pumps for each trial (Lejuez et al., 2002). Greater risk taking on the BART has been related to greater real-life risk taking as well as heightened ventral striatum and PFC activation (e.g., Hanson et al., 2014; Qu et al., 2015b). The second is risky exploration, which is measured by the within-person variation of the number of pumps before cash-outs (i.e., standard deviation in pump behavior across the task). The standard deviation of the number of pumps represents how spread participants’ risk-taking behavior is. For example, some individuals try a wide range of possibilities in the BART task, cashing out the money at different number of pumps across trials (i.e., having a large standard deviation of number of pumps). In contrast, other individuals may not explore this risky context and cash out the money at the fairly similar number of pumps (i.e., having a small standard deviation of number of pumps). Therefore, the standard deviation of number of pumps serves as a meaningful behavioral index of their risky exploration, with greater within-person variation of risk-taking behavior indicating greater risky exploration. A similar index has been used in prior research (e.g., Congdon et al., 2013; Goldenberg et al., 2017), and related to more real-life risk tendencies as well as greater white matter development in the brain.

#### fMRI Data Acquisition and Analysis

Imaging data were collected using a 3.0 Tesla Siemens Trio MRI scanner. The BART consisted of T2*-weighted echoplanar images (EPIs; slice thickness, 4 mm; 34 slices; TR = 2,000 ms; TE = 30 ms; flip angle = 90°; matrix = 64 × 64; FOV = 200 mm; voxel size 3 × 3 × 4 mm^3^). A T2*weighted, matched-bandwidth (MBW), high-resolution, anatomical scan and magnetization-prepared rapid-acquisition gradient echo (MPRAGE) scan were acquired for registration purposes (TR: 2.3; TE: 2.1; FOV: 256; matrix: 192 × 192; sagittal plane; slice thickness: 1 mm; 160 slices). The orientation for the MBW and EPI scans was oblique axial to maximize brain coverage.

Neuroimaging data were preprocessed using the FSL FMRIBs Software Library (FSL v6.0^1^). For each participant, motion correction was performed using MCFLIRT (Jenkinson et al., 2002). Spatial smoothing was applied using a Gaussian kernel of full width at half maximum (FWHM) of 6 mm. Following suggestions by Popescu et al. (2012), high-pass temporal filtering was then conducted with a filter width of 128 s (Gaussian-weighted least-squares straight line fitting, with sigma = 64.0 s) and images were skull-stripped using FSL’s Brain Extraction Tool (BET; Smith, 2002). Each functional image was registered to standard Montreal Neurological Institute (MNI) 2 mm brain through T2- and T1-weighted structural images using FLIRT (Jenkinson and Smith, 2001; Jenkinson et al., 2002). An individual level ICA denoising procedure was also conducted using MELODIC (Beckmann and Smith, 2004) in conjunction with an automated signal classification toolbox (classifier NP-threshold = 0.3) to remove motion- and physiological-related artifact (Tohka et al., 2008).

Each participant’s first level model was performed using Statistical Parametric Mapping (SPM8; Wellcome Department of Cognitive Neurology, Institute of Neurology, London, UK). Each trial was convolved with the canonical hemodynamic response function. High-pass temporal filtering with a cut-off of 128 s was applied to remove low-frequency drift in the time series. Serial autocorrelations were estimated with a restricted maximum likelihood algorithm with an autoregressive model order of 1. In each person’s fixed-effects first level model, multiple regressors were applied to separate different events: risk-taking decisions (i.e., pumps), receipt of rewards (i.e., cash outs), and receipt of negative outcome (i.e., explosions). Following prior studies, we only analyzed pumps on balloons that did not explode (i.e., pumps on each balloon prior to cash-out), because pumps on the explosion trials were necessarily constrained (Lejuez et al., 2002); these two types of trials were modeled separately. To test the linear relationship between brain activation and the magnitude of risks, each trial was modeled with a parametric modulator. The parametric modulator represented the pump number for each individual pump within a balloon, which was mean centered within participants. By parametrically modulating the level of pumps, we were able to examine whether neural regions show increasing activation as the level of risk increases. Null events, consisting of the jittered intertrial intervals, were not explicitly modeled and therefore constituted an implicit baseline. Because our main interest is neural responses during risk taking, we thus focus on the neural responses when participants engage in risky behavior.

Random effect analyses were conducted by submitting the individual subject contrasts to the second level for group analyses. Group analyses were conducted using GLMflex. GLMflex uses partitioned error terms, corrects for variance-covariance inequality, removes outliers and sudden changes in brain activation, and analyzes all voxels that have data^2^. To investigate whether American and Chinese participants show different neural activity during risk taking, we conducted a whole-brain, two-sample *t*-test (Americans-Chinese) analyses to explore neural regions that show differential activation across the two groups. To explore whether independent self-construal plays a similar or different role in American and Chinese participants, we conducted whole brain moderation analyses by regressing a cultural group × independent self-construal interaction term on neural activation during risk taking, controlling for the main effect of cultural group and independent self-construal. Findings from this whole-brain regression reflect the regions of the brain showing differential links with independent self-construal between American and Chinese participants.

To correct for multiple comparisons, we conducted a Monte Carlo simulation implemented using 3dClustSim in the software package AFNI^3^. We estimated the intrinsic smoothness of the data using the -acf option in 3dFWHMx. The results of the simulation indicated a voxel-wise threshold of *p* < 0.001 combined with a minimum cluster size of 25 voxels for two-sample *t*-test and 27 voxels for whole-brain moderation analysis. This joint voxel-wise and cluster-size threshold corresponds to *p* < 0.05, Family Wise Error (FWE) corrected. In order to take into account potential sex differences in risky exploration and neural processes during risk taking (e.g., Byrnes et al., 1999; Felton et al., 2003; Lee et al., 2009), participants’ sex was controlled for in all behavioral and fMRI analyses. However, behavioral and neuroimaging analyses without controlling for sex yield identical results.

## Results

### Group-Level Differences in Risky Exploration on the Behavioral Task

**Figure 1:**
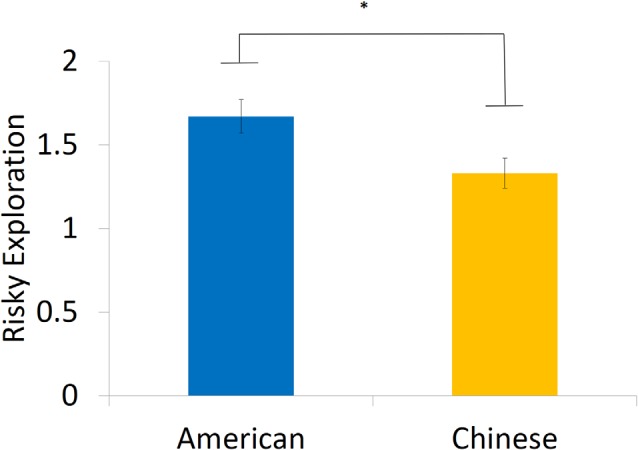
American participants showed significantly greater risky exploration compared with their Chinese counterparts. *Note*: risky exploration was measured by within-person variation of risk taking. *p < 0.05.

We first investigated overall performance on the BART in American and Chinese participants. American and Chinese participants did not differ in the average number of pumps before cash-outs on the task (Americans: *M* = 5.55, SD = 0.66, range = 2.81–8.18; Chinese: *M* = 5.44, SD = 0.88, range = 3.25–7.83; *β* = 0.06, *t* = 0.38, *p* = 0.704) or in the number of explosions they experienced (Americans: *M* = 9.55, SD = 2.70; Chinese: *M* = 9.21, SD = 2.81;* β* = 0.03, *t* = 0.24, *p* = 0.815). However, we did find differences in risky exploration, such that American participants exhibited greater within-person variation in risk taking (i.e., standard deviation in pumping behavior; *M* = 1.67, SD = 0.46) than did their Chinese counterparts (*M* = 1.33, SD = 0.43;* β* = 0.36, *t* = 2.55, *p* = 0.014, Cohen’s *d* = 0.76, 95% CI of the difference = [0.08, 0.61]), suggesting that American individuals are more likely to explore in a risky context (Figure 1).

We further conducted follow-up analyses to examine when the cultural difference in risky exploration emerges (i.e., do Chinese participants show lower variability from the beginning or do cultural differences emerge across the task?). To answer this question, we divided the 24 trials into four segments with six trials in each segment (i.e., trials 1–6; trials 7–12; trials 13–18; trials 19–24). We calculated within-person standard deviation in number of pumps for each segment and examined if American and Chinese participants differ across these four segments. We found that there is no difference in American and Chinese participants’ risky exploration in the first segment, *t* = 0.31, *p* = 0.758. However, cultural differences emerge in risky exploration after the first segment (second segment, *t* = 2.34, *p* = 0.024; third segment, *t* = 2.16, *p* = 0.036; fourth segment, *t* = 1.77, *p* = 0.084). These results suggest that American and Chinese participants do not differ in their risky exploration when they start the task, but as they experience the task, Chinese participants become more conservative and less likely to explore the upper and lower boundaries in later trials compared to American participants.

The two groups did not differ in the total points received on the BART (Americans: *M* = 76.14, SD = 10.48; Chinese: *M* = 75.42, SD = 8.69;* β* = 0.07, *t* = 0.48, *p* = 0.635), suggesting that they obtained the same outcome on the task, despite exploring the task in different ways. Group-level differences in performance on the BART and associations among them are presented in Tables s 1, 2.

**Table 1:** Descriptive statistics for independent self-construal and performance on the Balloon Analogue Risk Task (BART) in American and Chinese international students.

	American	Chinese	Cohen’s *d*
Independent self-construal	4.88 (0.48)	5.01 (0.62)	0.23
Average number of pumps before cash-outs	5.55 (0.66)	5.44 (0.88)	0.14
Within-person standard deviation in pumping behavior	1.67 (0.46)_a_	1.33 (0.43)_b_	0.76
Number of explosions	9.55 (2.70)	9.21 (2.81)	0.12
Total points received	76.14 (10.48)	75.42 (8.69)	0.07

*Note: different letter subscripts indicate a significant (*p* < 0.05) difference between the two groups*.

**Table 2:** Associations between independent self-construal and performance on the BART in American and Chinese international students.

	1	2	3	4	5
1. Independent self-construal	–	0.16	0.44*	0.32	−0.23
2. Average number of pumps before cash-outs	−0.14	–	0.01	0.85***	0.10
3. Within-person standard deviation in pumping behavior	−0.27	−0.11	–	0.09	0.00
4. Number of explosions	−0.14	0.64**	0.42*	–	−0.42*
5. Total points received	0.00	−0.09	−0.52*	−0.82***	–

Note: correlations for the American sample are presented in the lower triangle; those for the Chinese sample are presented in the upper triangle. *p < 0.05; **p < 0.01; ***p < 0.001.

### Group-Level Differences in Neural Activation during Risk Taking

At the neural level, we first investigated neural regions recruited as participants take increasing risks (i.e., during pumps) across the whole sample. To this end, we conducted a whole brain, one-sample *t*-test, controlling for cultural group. Neural regions that show increased activation during risk taking across participants are presented in Table 3. In whole brain, two-sample *t*-test analyses, we examined whether American and Chinese participants show different neural patterns during risk taking. No neural regions showed differential activation during risk taking between the two groups.

**Table 3:** Brain regions that showed activation when participants take increasing risks across the whole sample.

Anatomical Region	*x*	*y*	*z*	*t*	*k*
Positive activation					
ACC	8	22	32	9.26	5,831^a^
Right precentral gyrus	32	−8	56	5.93	^a^
Right postcentral gyrus	−40	−24	52	7.95	^a^
Left superior frontal gyrus	−20	−8	58	5.89	^a^
Right superior frontal gyrus	24	−8	58	7.49	^a^
Left insula	−34	22	8	7.43	1,113
Right insula	40	22	2	9.96	1,475
Right thalamus	8	−28	−2	9.45	2,813^b^
Left ventral striatum	−8	8	2	8.18	^b^
Right ventral striatum	10	8	2	7.37	^b^
Right middle frontal gyrus	32	46	24	4.17	57
Negative activation					
VLPFC	−52	24	26	−6.13	4,175^c^
Middle orbital gyrus	0	54	−6	−6.37	^c^
Rectal gyrus	2	42	−16	−7.83	^c^
Left middle frontal gyrus	−28	24	50	−6.10	^c^
Left postcentral gyrus	−32	−28	58	−7.11	^c^
Left amygdala	−16	−8	−16	−4.17	^c^
Right amygdala	16	−8	−16	−3.89	^c^
Right middle frontal gyrus	30	24	50	−4.70	741

*Note: x, y, and z refer to MNI coordinates; t refers to the t-score at those coordinates (local maxima); k refers to the number of voxels in each significant cluster. ACC, Anterior cingulate cortex. VLPFC, ventrolateral prefrontal cortex. Regions with the same superscript signify they belong to the same cluster of activation*.

### Independent Self-Construal and Risky Exploration by Cultural Group

Next, we examined whether American and Chinese participants differ in their self-reported independent self-construal. In line with the voluntary settlement hypothesis (Kitayama et al., 2006, 2014; Kitayama and Park, 2010), which suggests that voluntary immigrants moving to the frontiers (e.g., foreign countries) have greater independent self-construal than those who choose to stay in their original countries, Chinese participants in our study (i.e., self-motivated Chinese international students) reported similar level of independent self-construal (*M* = 5.01, SD = 0.48) compared to their American counterparts (*M* = 4.88, S*D* = 0.62), *β* = 0.13, *t* = 0.86, *p* = 0.397. The associations between independent self-construal and performance on the BART are presented in Table 2.

To explore whether independent self-construal plays a similar or differential role in risky exploration between American and Chinese participants, we conducted a cultural group × independent self-construal moderation analysis on participants’ risky exploration. We found a main effect of culture, *β* = 0.37, *t* = 2.68, *p* = 0.011, but no main effect of participants’ independent self-construal, *β* = 0.03, *t* = 0.23, *p* = 0.820. Importantly, there was a significant cultural group × independent self-construal interaction effect, *β* = 0.32, *t* = 2.26, *p* = 0.029. Follow-up analyses within each cultural group indicated that greater endorsement of independent self-construal was positively associated with greater risky exploration in Chinese participants, *β* = 0.46, *t* = 2.34, *p* = 0.029, but not in American participants, *β* = −0.26, *t* = −1.23, *p* = 0.235. Together, these findings suggest that independent self-construal plays a more salient role in Chinese participants’ risky exploration, such that those who had a more Western self-construal (i.e., independent self-construal) were more likely to behave like their American counterparts during risk taking (i.e., show greater risky exploration).

### Independent Self-Construal and Neural Activity During Risk Taking by Cultural Group

**Figure 2:**
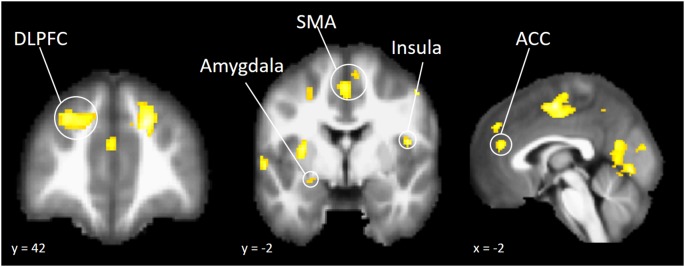
Brain regions that showed differential association with independent self-construal in American and Chinese participants.

To explore whether independent self-construal plays a different role in neural activation during risk taking in American and Chinese participants, we conducted a whole brain moderation analysis. The cultural group × independent self-construal interaction term was regressed onto brain activation during risk taking, controlling for the main effect of cultural group and independent self-construal. The results indicated that independent self-construal was differentially associated with American and Chinese participants’ neural activity in several regions of the cognitive control system, including DLPFC and ACC, as well as regions involved in affective salience, such as the AI and amygdala (Table 4). The brain map is presented in Figure 2 and is available on NeuroVault (see http://neurovault.org/collections/DBLXUJZI/).

**Table 4:** Brain regions that showed differential association with independent self-construal in American and Chinese participants.

Anatomical Region	*x*	*y*	*z*	*t*	* k*
Left DLPFC	−38	34	22	3.42	430
Right DLPFC	22	38	38	3.45	245
ACC	−2	46	18	3.74	50
Right superior medial gyrus	4	54	32	3.81	138
SMA	−2	−6	58	4.55	1,251
Right VLPFC	42	30	18	3.68	71
Right insula	44	−2	16	4.08	34
Left putamen	−32	−2	4	3.68	199
Left amygdala	−26	−2	−12	3.39	44
Left precentral gyrus	−36	−8	56	4.61	214
Left superior temporal gyrus	−60	−4	−2	4.39	205
Right superior temporal gyrus	54	−16	−2	3.87	81
Left middle temporal gyrus	−44	−58	2	4.32	479^a^
Left middle occipital gyrus	−40	−78	8	3.84	^a^
Right middle temporal gyrus	56	−46	−4	4.34	505
Left precuneus	−8	−74	48	3.63	77
Right precuneus	10	−74	48	3.85	764
Left cerebelum	−18	−60	−8	4.19	250
Right cerebelum	16	−60	−10	4.16	2,086^b^
Right fusiform gyrus	28	−50	−6	4.56	^b^
Right precuneus	20	−56	22	4.12	^b^
Right calcarine gyrus	10	−82	8	3.57	^b^

*Note: x, y, and z refer to MNI coordinates; t refers to the t-score at those coordinates (local maxima); k refers to the number of voxels in each significant cluster. DLPFC, dorsolateral prefrontal cortex. VLPFC, ventrolateral prefrontal cortex. ACC, Anterior cingulate cortex. SMA, supplementary motor area. Regions with the same superscript signify they belong to the same cluster of activation*.

**Figure 3:**
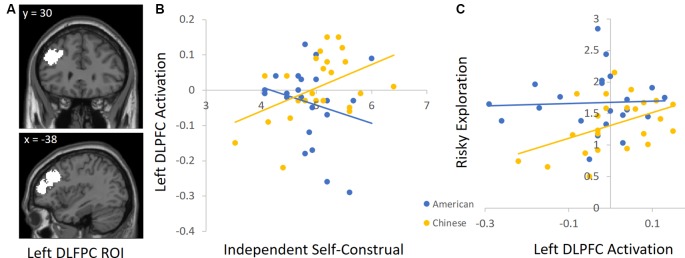
**(A)** The left dorsolateral prefrontal cortex (DLPFC) ROI derived from the Neurosynth platform. **(B)** Higher independent self-construal was associated with greater left DLPFC activation in Chinese (but not American) participants. **(C)** Greater left DLPFC activation was related to greater risky exploration in Chinese (but not American) participants.

**Figure 4:**
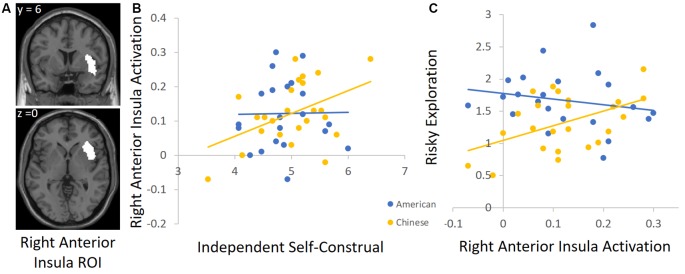
**(A)** The right anterior insula (AI) ROI derived from the Neurosynth platform. **(B)** Higher independent self-construal was associated with greater right AI activation in Chinese (but not American) participants. **(C)** Greater right AI activation was related to greater risky exploration in Chinese (but not American) participants.

To unpack the cultural group × independent self-construal interaction, we conducted follow-up analyses by using independent ROIs to examine the association between independent self-construal and neural activation in each cultural group separately. For descriptive purposes, we focused on the DLPFC and AI, but the results are similar for the other regions identified in Table 4. For the DLPFC ROI, we used the automated meta-analysis with the term “dorsolateral prefrontal” from the association test map (*q* < 0.1 FDR-corrected) in the NeuroSynth database^4^. Given that other regions emerged in this search (e.g., right DLPFC), we further restricted the cluster to the left DLPFC (see Figure 3A for this ROI). To define the insula ROI, we used the automated meta-analysis with the term “anterior insula” from the association test map (*q* < 0.1 FDR-corrected) in the NeuroSynth database^5^ and restricted the cluster to the right anterior insula (see Figure 4A for this ROI).

We used these ROIs to extract parameter estimates of signal intensity from the DLPFC and AI during risk taking in each cultural group. We then conducted correlation analyses in SPSS to examine the association between independent self-construal and neural activation within American and Chinese participants separately. Consistent with the behavioral analyses, greater independent self-construal was associated with greater DLFPC activity during risk taking in Chinese participants (*r* = 0.44, *p* = 0.036), but the association was not significant in American participants (*r* = −0.23, *p* = 0.308; Figure 3B). Similarly, greater independent self-construal was associated with greater AI activity during risk taking in Chinese participants (*r* = 0.43, *p* = 0.040), but the association was not significant in American participants (*r* = 0.02, *p* = 0.936; Figure 4B).

Next, we examined how DLPFC and insula activation during risk taking are related to risky exploration on the BART in American and Chinese participants. We used the same ROIs and conducted correlation analyses with participants’ risky exploration in SPSS. Greater DLFPC activity was associated with greater risky exploration in Chinese participants (*r* = 0.45, *p* = 0.032), but not in American participants (*r* = 0.13, *p* = 0.568; Figure 3C). Similarly, greater AI activity was associated with greater risky exploration in Chinese participants (*r* = 0.52, *p* = 0.011), but not in American participants (*r* = −0.21, *p* = 0.358; Figure 4C). Together, these findings suggest that Chinese (but not American) participants who reported greater independent self-construal showed greater activation in regions supporting cognitive control and affective processes when engaging in risk taking, and such heightened neural activity was related to greater risky exploration.

## Discussion

Although prior research examines cross-cultural differences in self-reported risk-taking behavior or risk preference (Weber and Hsee, 1998; Greenberger et al., 2000), little is known about the role of culture in the motivation of risk taking and the underpinning neural correlates as individuals acculturate to a new culture. The present study addressed this gap by conceptualizing culture at two levels: culture as nationality/group membership (i.e., American vs. Chinese) and culture as individual beliefs/orientation (i.e., independent self-construal). Consistent with the emphasis on adventure and risk exploration in American culture (Doyle, 1999), our findings suggest that American individuals show greater risky exploration in a risk-taking task than Chinese individuals. With regard to individuals’ cultural orientation, we find that independent self-construal plays a differential role in American and Chinese international students. While independent self-construal is not related to American individuals’ behavioral performance and neural correlates of risk taking, Chinese students who report greater independent self-construal engage in greater risky exploration and recruit greater activation in the cognitive control and affective systems when engaging in risk taking. Taken together, our findings suggest that culture modulates the neural correlates of risk taking, highlighting the importance of examining the role of culture in neural functions as both group membership and individual orientation to better understand how individuals acculturate to a new culture.

It has been documented that American culture places a strong emphasis on adventure and risk exploration (Doyle, 1999). Therefore, American individuals are culturally motivated to explore in a risky context. In contrast, such appreciation of exploration is not evident in East Asian countries (King and Bond, 1985; Ho, 1986; Chen et al., 1992, 1998). Prior studies on cultural differences in risk taking and risk performance relied on self-reported measures and are unable to assess the extent to which individuals explore risky contexts in a dynamic manner. Using a behavioral task, the present study is the first to quantify individuals’ risky exploration. Consistent with cultural differences in the emphasis on exploration, American individuals showed greater risky exploration in this behavioral task compared to their Chinese counterparts. Notably, our follow-up analyses suggest that American and Chinese participants did not differ in their risky exploration when they start the task. However, as they experience the task, Chinese participants become more conservative and less likely to explore the upper and lower boundaries in later trials compared to American participants. This finding provides novel insights into cultural differences in risky exploration, highlighting that such cultural differences emerge across the task rather than from the beginning. Despite difference in how the two groups explored the task environment, they did not differ in mean level of risk taking or in the number of explosions. Thus, American participants had a larger and more variable boundary by which they pumped, but the average number of pumps evened out between the two cultural groups. Thus, when participants were instructed to take risks to get as many points as possible, both American and Chinese participants performed similarly with regard to their overall risk taking and earned a similar number of points, but they got there using different behavioral strategies, suggesting that risky exploration reflects meaningful cultural differences.

In addition to examining cultural differences at the group level, the present study investigated the role of individuals’ cultural orientation and whether it plays a similar role across the two groups. Specifically, we focused on independent self-construal, a way of describing or defining the self that emphasizes unique attributes and qualities (Markus and Kitayama, 1991; Kitayama et al., 2007; Cross et al., 2011). Although American individuals are hypothesized to show greater independent self-construal than Chinese individuals, researchers also suggest that voluntary immigrants who move to the frontiers (e.g., those who move to a foreign country) may report greater independent self-construal than those who choose to stay in their original countries (Kitayama et al., 2006, 2014; Kitayama and Park, 2010). In line with this idea, Chinese participants in our study, who are self-motivated Chinese students studying in the United States, reported a similar level of independent self-construal as American participants. This finding is also consistent with recent evidence that self-motivated Chinese immigrants may show neural patterns that are different from those who stay in China (Chen et al., 2013).

While American and Chinese participants reported similar levels of independent self-construal, the role of independent self-construal functioned differently across the two groups at both the behavioral and neural levels. Chinese participants who reported greater independent self-construal tended to explore to a greater extent in a risky context, suggesting that the greater focus on personal traits, abilities, values, and preferences, may support risky exploration. It is possible that the heightened emphasis on internal attributes and preferences makes them more willing to explore the environment, which is not only reflected in their voluntary migration to the United States for education but also reflected in their greater exploratory behavior in risk-taking contexts. However, such association is not evident in American participants. It is possible that American individuals are already immersed in an independent culture, and individual differences in endorsement of such cultural norms may not be salient in guiding their behavior and predicting culturally valued risky exploration. More importantly, past research has suggested that cultural differences that are observed at the country or group level may not always be reduced to individual differences (Na et al., 2010). For example, even if East Asians and Westerners consistently differ in social orientation measures at the group level, the individual-level correlations of these measures are low. This suggests that group-level difference is independent of individual-level correlations, and statistically, the individual-level correlations can be positive, zero, or negative, in the context of group-level difference. Similarly, in our case, although Americans are expected to show greater independent self-construal and risky exploration compared to East Asians at the group level (i.e., between-group difference), it does not indicate that independent self-construal and risky exploration would be associated within Americans (i.e., within-group association).

Independent self-construal also played a differential role at the neural level in American and Chinese international students. Specifically, Chinese participants who reported greater independent self-construal recruited greater activation when engaging in risk taking in several regions supporting cognitive control (e.g., DLPFC), as well as affective salience (e.g., AI), and greater activation in these regions was related to greater risky exploration. This finding may seem surprising at first glance, given that greater activation in the cognitive control system is often related to lower risk-taking behavior (Fecteau et al., 2007; Knoch and Fehr, 2007; Cohen and Lieberman, 2010; Telzer et al., 2013). However, it is possible that Chinese participants who hold more independent cultural beliefs tended to regulate their behavior in the risk-taking context to use a more exploratory strategy. That is, greater activation in their cognitive control system reflects their greater tendency to exert control to explore the novel environment. Moreover, past research suggests that the AI and amygdala are involved in detecting affective salience and the AI is a neural hub integrating affective and cognitive signals (e.g., Anderson and Phelps, 2001; Fitzgerald et al., 2006; Craig, 2009; Menon and Uddin, 2010; Smith et al., 2014; van Duijvenvoorde et al., 2015). Greater activation in these neural regions indicates that Chinese participants with more independent cultural beliefs may engage in greater integration of their emotional and regulatory processes during risk taking. This finding extends our prior understanding of cognitive control and affective salience, highlighting that neural regions may function differently for individuals who attempt to acculturate to a new environment. For American participants, similar to its association with behavioral performance, independent self-construal was not related to neural correlates as they take increasing risks.

The current study highlights the role of culture in the neural correlates of risk taking and links to risky exploration. One novel feature was to conceptualize culture at both the group level and individual level by comparing American students with Chinese international students who are acculturating to American culture. In future research, it is also important to compare American individuals with Chinese individuals who reside in China or American individuals who move to East Asian cultures in order to investigate potential differences. Moreover, we only focus on independent self-construal as individual-level cultural orientation. Future studies are needed to examine interdependent self-construal, which is more frequent in East Asian cultures. For example, it will be interesting to examine the role of interdependent self-construal in American individuals’ behavioral performance and neural correlates of risky exploration as they become acculturated to East Asian cultures. Also, in the current study, we recruited Chinese international students who had moved to the United States within 1 year of their arrival. To capture a full range of acculturation processes, future studies should recruit a sample with large variation in immigrant status (e.g., from newly immigrated to second generation). It is important to examine other dimensions of individual differences in acculturation, such as time spent in the United States, to have a greater understanding of the behavioral and neural processes of risky exploration.

In addition, although this study quantified individuals’ risky exploration, it is important for future research to link behavioral performance and neural correlates of risky exploration with real-life outcomes (Chen et al., 2015). On the one hand, it is possible that risky exploration may be related to negative health-related outcomes, such as smoking, drinking, and drug use. On the other hand, it is also possible that risky exploration can serve adaptive functions and contribute to behavior associated with positive outcomes, such as entrepreneurship and cross-cultural communication. Indeed, risky exploration may reflect learning and adaptation to one’s changing environment and is associated with lower riskiness ratings of real-world risky behaviors (Goldenberg et al., 2017). Moreover, following most studies, participants in the present study completed the behavioral task alone. However, real-life risk taking also occurs in social contexts and needs to receive attention. Indeed, prior research has demonstrated the impacts of parents and peers on adolescents’ risk taking and underlying neural mechanisms (e.g., Chein et al., 2011; Qu et al., 2015a, 2016; Telzer et al., 2015). Given the different emphasis of significant others in individuals’ self-construal in independent and interdependent cultures, future research is needed to investigate how parents and friends affect the neural basis of risky exploration across cultures, which can provide more insights into the role of social contexts in risky exploration.

The current study has several limitations, pointing to directions for future research. First, caution should be taken when interpreting the findings given the small sample size. For example, in our study, the correlation between self-construal and DLPFC activation was significant in Chinese participants, but not in American participants. However, both effects were moderate in their effect size. Given the small samples, it is important to test the robustness of this finding using a large sample; perhaps with a larger sample, the association in Chinese and American participants would yield more robust effects. Therefore, future behavioral and neuroimaging studies are needed to replicate the findings in a larger sample size. Moreover, given that the fMRI scans for the Chinese sample in our study only span 2 months, individual variation in the time they live in the U.S. is small, which limits our ability to explore the associations between the time spent in the U.S. and independent self-construal, risky exploration, as well as neural reactivity. Future research needs to recruit participants with a wider range of time living in the U.S. to explore individual differences within the Chinese sample with attention to the impact of the time on psychological and neural processes. In addition, the current study only focuses on cultural differences in univariate neural activation during risky taking. To fully understand the role of culture in neural underpinnings of risky exploration, it is important for future research to take a pattern-focused approach by employing the representational similarity analysis (RSA; Kriegeskorte et al., 2008). This approach allows researchers to test how neural representations during risk taking are similar by focusing on signal variations of neural response across voxels (i.e., multi-voxel pattern), rather than simply changes in the overall fMRI magnitude between cultural groups (i.e., univariate neural activation).

In conclusion, using a behavioral risk-taking task, the current study demonstrated that American individuals showed greater risky exploration compared to their Chinese counterparts. Moreover, Chinese international students who reported greater independent self-construal engaged in greater risky exploration and recruited more activation in regions of the cognitive control and affective systems when engaging in risk taking. Taken together, our findings suggest that culture modulates individuals’ risk exploration, and more importantly, culture as group membership and individual orientation may interact with each other and relate to neural systems supporting cognitive control and affective salience. These findings make a novel contribution to a growing literature on the cultural neuroscience of acculturation, highlighting the importance of examining the role of culture as both group membership and individual orientation in behavioral and neural processes, especially for those who are acculturating to a new environment.

## Ethics Statement

In conducting this research, we complied with the American Psychological Association ethical standards in the treatment of the participants. The research was approved by the Institutional Review Boards of the University of Illinois. The current report is not being considered elsewhere for publication.

## Author Contributions

YQ, LL, and ET designed the research. YQ and LL performed the research. YQ analyzed the data. YQ and ET wrote the article.

## Conflict of Interest Statement

The authors declare that the research was conducted in the absence of any commercial or financial relationships that could be construed as a potential conflict of interest.

## Footnotes

^1^www.fmrib.ox.ac.uk/fsl

^2^http://mrtools.mgh.harvard.edu/index.php/GLM_Flex

^3^http://afni.nimh.nih.gov/afni/

^4^http://www.neurosynth.org. Accessed March 29, 2019.

## References

[B1] Aklin W. M. Lejuez C. W. Zvolensky M. J. Kahler C. W. Gwadz M. (2005). Evaluation of behavioral measures of risk taking propensity with inner city adolescents. Behav. Res. Ther. 43, 215–228. 10.1016/j.brat.2003.12.007 15629751

[B2] Almeida J. Johnson R. M. Matsumoto A. Godette D. C. (2012). Substance use, generation and time in the United States: the modifying role of gender for immigrant urban adolescents. Soc. Sci. Med. 75, 2069–2075. 10.1016/j.socscimed.2012.05.016 22727651 PMC3461090

[B3] Anderson A. K. Phelps E. A. (2001). Lesions of the human amygdala impair enhanced perception of emotionally salient events. Nature 411, 305–309. 10.1038/35077083 11357132

[B4] Beckmann C. F. Smith S. M. (2004). Probabilistic independent component analysis for functional magnetic resonance imaging. IEEE Trans. Med. Imaging 23, 137–152. 10.1109/TMI.2003.822821 14964560

[B5] Brindis C. Wolfe A. L. McCarter V. Ball S. Starbuck-Morales S. (1995). The associations between immigrant status and risk-behavior patterns in Latino adolescents. J. Adolesc. Health 17, 99–105. 10.1016/1054-139x(94)00101-j 7495832

[B6] Brislin R. W. (1980). “Translation and content analysis of oral and written materials,” in Handbook of Cross-Cultural Psychology Vol. 2. Methodology, eds Triandis H. C. Berry J. W. (Boston, MA: Allyn and Bacon), 389–444.

[B7] Byrnes J. P. Miller D. C. Schafer W. D. (1999). Gender differences in risk taking: a meta-analysis. Psychol. Bull. 125, 367–383. 10.1037/0033-2909.125.3.367

[B8] Camus M. Halelamien N. Plassmann H. Shimojo S. O’Doherty J. Camerer C.. (2009). Repetitive transcranial magnetic stimulation over the right dorsolateral prefrontal cortex decreases valuations during food choices. Eur. J. Neurosci. 30, 1980–1988. 10.1111/j.1460-9568.2009.06991.x 19912330

[B9] Chein J. Albert D. O’Brien L. Uckert K. Steinberg L. (2011). Peers increase adolescent risk taking by enhancing activity in the brain’s reward circuitry. Dev. Sci. 14, F1–F10. 10.1111/j.1467-7687.2010.01035.x 21499511 PMC3075496

[B12] Chen X. Hastings P. D. Rubin K. H. Chen H. Cen G. Stewart S. L. (1998). Child-rearing attitudes and behavioral inhibition in Chinese and Canadian toddlers: a cross-cultural study. Dev. Psychol. 34, 677–686. 10.1037/0012-1649.34.4.677 9681259

[B10] Chen P.-H. A. Heatherton T. F. Freeman J. B. (2015). “Brain-as-predictor approach: an alternative way to explore acculturation processes,” in Neuroscience in Intercultural Contexts, eds Warnick J. E. Landis D. (New York, NY : Springer), 143–170.

[B13] Chen X. Rubin K. H. Sun Y. (1992). Social reputation and peer relationships in Chinese and Canadian children: a cross-cultural study. Child Dev. 63, 1336–1343. 10.1111/j.1467-8624.1992.tb01698.x

[B11] Chen P.-H. A. Wagner D. D. Kelley W. M. Powers K. E. Heatherton T. F. (2013). Medial prefrontal cortex differentiates self from mother in Chinese: evidence from self-motivated immigrants. Cult. Brain 1, 3–15. 10.1007/s40167-013-0001-5

[B14] Cheon B. K. Im D. M. Harada T. Kim J. S. Mathur V. A. Scimeca J. M.. (2011). Cultural influences on neural basis of intergroup empathy. Neuroimage 57, 642–650. 10.1016/j.neuroimage.2011.04.031 21549201

[B15] Chiu C.-P. Tlustos S. J. Walz N. C. Holland S. K. Eliassen J. C. Bernard L.. (2012). Neural correlates of risky decision making in adolescents with and without traumatic brain injury using the balloon analog risk task. Dev. Neuropsychol. 37, 176–183. 10.1080/87565641.2011.632796 22339229 PMC3707800

[B16] Cohen J. R. Lieberman M. D. (2010). “The common neural basis of exerting self-control in multiple domains,” in Self Control in Society, Mind and Brain, eds Hassin R. R. Ochsne K. N. Trope Y. (New York: Oxford University Press), 141–160.

[B17] Congdon E. Bato A. A. Schonberg T. Mumford J. A. Karlsgodt K. H. Sabb F. W.. (2013). Differences in neural activation as a function of risk-taking task parameters. Front. Neurosci. 7:173. 10.3389/fnins.2013.00173 24137106 PMC3786224

[B18] Coon H. M. Kemmelmeier M. (2001). Cultural orientations in the United States: (Re) examining differences among ethnic groups. J. Cross Cult. Psychol. 32, 348–364. 10.1177/0022022101032003006

[B19] Craig A. D. (2009). How do you feel—now? The anterior insula and human awareness. Nat. Rev. Neurosci. 10, 59–70. 10.1038/nrn2555 19096369

[B20] Cross S. E. (1995). Self-construals, coping and stress in cross-cultural adaptation. J. Cross Cult. Psychol. 26, 673–697. 10.1177/002202219502600610

[B21] Cross S. E. Hardin E. E. Gercek-Swing B. (2011). The what, how, why and where of self-construal. Pers. Soc. Psychol. Rev. 15, 142–179. 10.1177/1088868310373752 20716643

[B22] Delgado M. Nystrom L. Fissell C. Noll D. Fiez J. (2000). Tracking the hemodynamic responses to reward and punishment in the striatum. J. Neurophysiol. 84, 3072–3077. 10.1152/jn.2000.84.6.3072 11110834

[B640] Doyle K. O. (1999). The Social Meanings of Money and Property: In Search of a Talisman. Thousand Oaks, CA: Sage.

[B23] Fecteau S. Knoch D. Fregni F. Sultani N. Boggio P. Pascual-Leone A. (2007). Diminishing risk-taking behavior by modulating activity in the prefrontal cortex: a direct current stimulation study. J. Neurosci. 27, 12500–12505. 10.1523/JNEUROSCI.3283-07.2007 18003828 PMC6673338

[B24] Felton J. Gibson B. Sanbonmatsu D. M. (2003). Preference for risk in investing as a function of trait optimism and gender. J. Behav. Finance 4, 33–40. 10.1207/s15427579jpfm0401_05

[B25] Fitzgerald D. A. Angstadt M. Jelsone L. M. Nathan P. J. Phan K. L. (2006). Beyond threat: amygdala reactivity across multiple expressions of facial affect. Neuroimage 30, 1441–1448. 10.1016/j.neuroimage.2005.11.003 16368249

[B26] Galván A. Hare T. Voss H. Glover G. Casey B. J. (2007). Risk-taking and the adolescent brain: who is at risk? Dev. Sci. 10, F8–F14. 10.1111/j.1467-7687.2006.00579.x 17286837

[B27] Galván A. Schonberg T. Mumford J. Kohno M. Poldrack R. A. London E. D. (2013). Greater risk sensitivity of dorsolateral prefrontal cortex in young smokers than in nonsmokers. Psychopharmacology 229, 345–355. 10.1007/s00213-013-3113-x 23644912 PMC3758460

[B28] Goldenberg D. Telzer E. H. Lieberman M. D. Fuligni A. J. Galván A. (2017). Greater response variability in adolescents is associated with increased white matter development. Soc. Cogn. Affect. Neurosci. 12, 436–444. 10.1093/scan/nsw132 27651539 PMC5390745

[B29] Goldstein R. Z. Volkow N. D. (2011). Dysfunction of the prefrontal cortex in addiction: neuroimaging findings and clinical implications. Nat. Rev. Neurosci. 12, 652–669. 10.1038/nrn3119 22011681 PMC3462342

[B740] Greenberger E. Chen C. Beam M. Whang S.-M. Dong Q. (2000). The perceived social contexts of adolescents’ misconduct: A comparative study of youths in three cultures. J. Res. Adolesc. 10, 365–388. 10.1207/SJRA1003_724336725

[B30] Hanson K. L. Thayer R. E. Tapert S. F. (2014). Adolescent marijuana users have elevated risk-taking on the balloon analog risk task. J. Psychopharmacol. 28, 1080–1087. 10.1177/0269881114550352 25237125 PMC8898087

[B31] Heatherton T. F. Wagner D. D. (2011). Cognitive neuroscience of self-regulation failure. Trends Cogn. Sci. 15, 132–139. 10.1016/j.tics.2010.12.005 21273114 PMC3062191

[B32] Heitmueller A. (2005). Unemployment benefits, risk aversion and migration incentives. J. Popul. Econ. 18, 93–112. 10.1007/s00148-004-0192-3

[B33] Ho D. Y. F. (1986). “Chinese pattern of socialization: a critical review,” in The Psychology of the Chinese People, ed. Bond M. H. (New York, NY: Oxford University Press), 1–37.

[B34] Jaeger D. A. Dohmen T. Falk A. Huffman D. Sunde U. Bonin H. (2010). Direct evidence on risk attitudes and migration. Rev. Econ. Stat. 92, 684–689. 10.1162/REST_a_00020

[B36] Jenkinson M. Bannister P. Brady M. Smith S. (2002). Improved optimization for the robust and accurate linear registration and motion correction of brain images. Neuroimage 17, 825–841. 10.1016/s1053-8119(02)91132-8 12377157

[B35] Jenkinson M. Smith S. (2001). A global optimisation method for robust affine registration of brain images. Med. Image Anal. 5, 143–156. 10.1016/s1361-8415(01)00036-6 11516708

[B37] King A. Y. C. Bond M. H. (1985). “The confucian paradigm of man: a sociological view,” in Chinese Culture and Mental Health, eds Tseng W. S. Wu D. Y. H. (San Diego, CA: Academic Press), 29–45.

[B39] Kitayama S. Duffy S. Uchida Y. (2007). “Self as cultural mode of being,” in Handbook of Cultulal Psychology, eds Kitayama S. Cohen D. (New York, NY: Guilford Press), 136–174.

[B40] Kitayama S. Ishii K. Imada T. Takemura K. Ramaswamy J. (2006). Voluntary settlement and the spirit of independence: evidence from Japan’s “Northern frontier”. J. Pers. Soc. Psychol. 91, 369–384. 10.1037/0022-3514.91.3.369 16938025

[B41] Kitayama S. King A. Yoon C. Tompson S. Huff S. Liberzon I. (2014). The dopamine D4 receptor gene (DRD4) moderates cultural difference in independent versus interdependent social orientation. Psychol. Sci. 25, 1169–1177. 10.1177/0956797614528338 24747168

[B38] Kitayama S. Park J. (2010). Cultural neuroscience of the self: understanding the social grounding of the brain. Soc. Cogn. Affect. Neurosci. 5, 111–129. 10.1093/scan/nsq052 20592042 PMC2894676

[B42] Kitayama S. Varnum M. E. W. Sevincer A. T. (2014). “The frontier: Voluntary settlement and cultural change,” in Culture Reexamined: Broadening Our Understanding of Social and Evolutionary Influences, ed. Cohen A. (Washington, DC: APA), 93–127.

[B43] Knoch D. Fehr E. (2007). Resisting the power of temptations: the right prefrontal cortex and self-control. Ann. N Y Acad. Sci. 1104, 123–134. 10.1196/annals.1390.004 17344543

[B44] Knutson B. Westdorp A. Kaiser E. Hommer D. (2000). fMRI visualization of brain activity during a monetary incentive delay task. Neuroimage 12, 20–27. 10.1006/nimg.2000.0593 10875899

[B45] Kriegeskorte N. Mur M. Bandettini P. (2008). Representational similarity analysis—connecting the branches of systems neuroscience. Front. Syst. Neurosci. 2:4. 10.3389/neuro.06.004.2008 19104670 PMC2605405

[B46] Lee T. M. C. Chan C. C. H. Leung A. W. S. Fox P. T. Gao J.-H. (2009). Sex-related differences in neural activity during risk taking: an fMRI study. Cereb. Cortex 19, 1303–1312. 10.1093/cercor/bhn172 18842666 PMC2677650

[B49] Lejuez C. W. Aklin W. M. Daughters S. Zvolensky M. Kahler C. Gwadz M. (2007). Reliability and validity of the youth version of the balloon analogue risk task (BART-Y) in the assessment of risk-taking behavior among inner-city adolescents. J. Clin. Child Adolesc. Psychol. 36, 106–111. 10.1080/15374410709336573 17206886

[B47] Lejuez C. W. Aklin W. M. Zvolensky M. J. Pedulla C. M. (2003). Evaluation of the balloon analogue risk task (BART) as a predictor of adolescent real-world risk-taking behaviors. J. Adolesc. 26, 475–479. 10.1016/s0140-1971(03)00036-8 12887935

[B48] Lejuez C. W. Read J. P. Kahler C. W. Richards J. B. Ramsey S. E. Stuart G. L.. (2002). Evaluation of a behavioral measure of risk taking: the balloon analogue risk task (BART). J. Exp. Psychol. Appl. 8, 75–84. 10.1037/1076-898x.8.2.75 12075692

[B50] Markus H. R. Kitayama S. (1991). Culture and the self: implications for cognition, emotion and motivation. Psychol. Rev. 98, 224–253. 10.1037/0033-295x.98.2.224

[B730] McCormick E. Telzer E. H. (2017). Adaptive adolescent flexibility: Neurodevelopment of decision-making and learning in a risky context. J. Cogn. Neurosci. 29, 413–423. 10.1162/jocn_a_01061 28129057 PMC5362273

[B51] Menon V. Uddin L. Q. (2010). Saliency, switching, attention and control: a network model of insula function. Brain Struct. Funct. 214, 655–667. 10.1007/s00429-010-0262-0 20512370 PMC2899886

[B52] Miller E. K. Cohen J. D. (2001). An integrative theory of prefrontal cortex function. Annu. Rev. Neurosci. 24, 167–202. 10.1146/annurev.neuro.24.1.167 11283309

[B53] Na J. Grossmann I. Varnum M. E. Kitayama S. Gonzalez R. Nisbett R. E. (2010). Cultural differences are not always reducible to individual differences. Proc. Natl. Acad. Sci. U S A 107, 6192–6197. 10.1073/pnas.1001911107 20308553 PMC2851996

[B54] Ojeda V. D. Patterson T. L. Strathdee S. A. (2008). The influence of perceived risk to health and immigration-related characteristics on substance use among Latino and other immigrants. Am. J. Public Health 98, 862–868. 10.2105/AJPH.2006.108142 18382009 PMC2374816

[B55] Popescu V. Battaglini M. Hoogstrate W. S. Verfaillie S. C. Sluimer I. C. van Schijndel R. A.. (2012). Optimizing parameter choice for FSL brain extraction tool (BET) on 3D T1 images in multiple sclerosis. Neuroimage 61, 1484–1494. 10.1016/j.neuroimage.2012.03.074 22484407

[B56] Prado G. Huang S. Schwartz S. J. Maldonado-Molina M. M. Bandiera F. de la Rosa M.. (2009). What accounts for differences in substance use among U.S.-born and immigrant hispanic adolescents?: results from a longitudinal prospective cohort study. J. Adolesc. Health 45, 118–125. 10.1016/j.jadohealth.2008.12.011 19628137 PMC3466101

[B57] Qu Y. Fuligni A. J. Galván A. Lieberman M. D. Telzer E. H. (2016). Links between parental depression and longitudinal changes in youths’ neural sensitivity to rewards. Soc. Cogn. Affect. Neurosci. 11, 1262–1271. 10.1093/scan/nsw035 27013103 PMC4967797

[B580] Qu Y. Fuligni A. J. Galván A. Telzer E. H. (2015a). Buffering effect of positive parent-child relationships on adolescent risk taking: A longitudinal neuroimaging investigation. Dev. Cogn. Neurosci. 15, 26–34. 10.1016/j.dcn.2015.08.005 26342184 PMC4639442

[B58] Qu Y. Galván A. Fuligni A. J. Lieberman M. D. Telzer E. H. (2015b). Longitudinal changes in prefrontal cortex activation underlie declines in adolescent risk taking. J. Neurosci. 35, 11308–11314. 10.1523/JNEUROSCI.1553-15.2015 26269638 PMC4532760

[B59] Salas-Wright C. P. Vaughn M. G. Schwartz S. J. Córdova D. (2016). An “immigrant paradox” for adolescent externalizing behavior? Evidence from a national sample. Soc. Psychiatry Psychiatr. Epidemiol. 51, 27–37. 10.1007/s00127-015-1115-1 26328521 PMC4724222

[B60] Schonberg T. Fox C. R. Mumford J. A. Congdon E. Trepel C. Poldrack R. A. (2012). Decreasing ventromedial prefrontal cortex activity during sequential risk-taking: an fMRI investigation of the balloon analog risk task. Front. Neurosci. 6:80. 10.3389/fnins.2012.00080 22675289 PMC3366349

[B61] Singelis T. M. (1994). The measurement of independent and interdependent self-construals. Pers. Soc. Psychol. Bull. 20, 580–591. 10.1177/0146167294205014

[B62] Singer T. Critchley H. D. Preuschoff K. (2009). A common role of insula in feelings, empathy and uncertainty. Trends Cogn. Sci. 13, 334–340. 10.1016/j.tics.2009.05.001 19643659

[B64] Smith S. M. (2002). Fast robust automated brain extraction. Hum. Brain Mapp. 17, 143–155. 10.1002/hbm.10062 12391568 PMC6871816

[B63] Smith A. R. Steinberg L. Chein J. (2014). The role of the anterior insula in adolescent decision making. Dev. Neurosci. 36, 196–209. 10.1159/000358918 24853135 PMC5544351

[B65] Telzer E. H. Fuligni A. J. Lieberman M. D. Galván A. (2013). The effects of poor quality sleep on brain function during risk taking in adolescence. Neuroimage 71, 275–283. 10.1016/j.neuroimage.2013.01.025 23376698 PMC3865864

[B66] Telzer E. H. Fuligni A. J. Lieberman M. D. Galván A. (2014). Neural sensitivity to eudaimonic and hedonic rewards differentially predict adolescent depressive symptoms over time. Proc. Natl. Acad. Sci. U S A 111, 6600–6605. 10.1073/pnas.1323014111 24753574 PMC4020059

[B67] Telzer E. H. Ichien N. I. Qu Y. (2015). Mothers know best: redirecting adolescent reward sensitivity to promote safe behavior during risk taking. Soc. Cogn. Affect. Neurosci. 10, 1383–1391. 10.1093/scan/nsv026 25759470 PMC4590537

[B68] Tohka J. Foerde K. Aron A. R. Tom S. M. Toga A. W. Poldrack R. A. (2008). Automatic independent component labeling for artifact removal in fMRI. Neuroimage 39, 1227–1245. 10.1016/j.neuroimage.2007.10.013 18042495 PMC2374836

[B69] van Duijvenvoorde A. C. K. Huizenga H. M. Somerville L. H. Delgado M. R. Powers A. Weeda W. D.. (2015). Neural correlates of expected risks and returns in risky choice across development. J. Neurosci. 35, 1549–1560. 10.1523/JNEUROSCI.1924-14.2015 25632132 PMC6795265

[B70] Van Leijenhorst L. Zanolie K. Van Meel C. S. Westenberg P. M. Rombouts S. A. R. B. Crone E. A. (2010). What motivates the adolescent? Brain regions mediating reward sensitivity across adolescence. Cereb. Cortex 20, 61–69. 10.1093/cercor/bhp078 19406906

[B71] Wang C. Oyserman D. Liu Q. Li H. Han S. (2013). Accessible cultural mind-set modulates default mode activity: evidence for the culturally situated brain. Soc. Neurosci. 8, 203–216. 10.1080/17470919.2013.775966 23485156

[B72] Wang F. Peng K. Chechlacz M. Humphreys G. W. Sui J. (2017). The neural basis of independence versus interdependence orientations: a voxel-based morphometric analysis of brain volume. Psychol. Sci. 28, 519–529. 10.1177/0956797616689079 28406379

[B73] Weber E. U. Hsee C. (1998). Cross-cultural differences in risk perception, but cross-cultural similarities in attitudes towards perceived risk. Manage. Sci. 44, 1205–1217. 10.1287/mnsc.44.9.1205

[B74] Wessel J. R. Conner C. R. Aron A. R. Tandon N. (2013). Chronometric electrical stimulation of right inferior frontal cortex increases motor braking. J. Neurosci. 33, 19611–19619. 10.1523/JNEUROSCI.3468-13.2013 24336725 PMC3858630

